# Calcified coccoid from Cambrian Miaolingian: Revealing the potential cellular structure of *Epiphyton*

**DOI:** 10.1371/journal.pone.0213695

**Published:** 2019-03-14

**Authors:** Xiyang Zhang, Mingyue Dai, Min Wang, Yong’an Qi

**Affiliations:** 1 School of Resources and Environment, Henan Polytechnic University, Jiaozuo, Henan, China; 2 State Key Laboratory of Palaeobiology and Stratigraphy, Nanjing Institute of Geology and Palaeontology and Center for Excellence in Life and Paleoenvironment, Chinese Academy of Sciences, Nanjing, Jiangsu, China; Columbia University, UNITED STATES

## Abstract

*Epiphyton*, *Renalcis*, and *Girvanella* are ubiquitous genera of calcified cyanobacteria/algae from Early Paleozoic shallow-marine limestones. One genus, *Epiphyton*, is characterized by a particular dendritic outline, and extensive research has revealed the morphology of calcified remains although little information on cellular structure is known. The mass occurrence of calcified *Epiphyton* in microbialites from Cambrian Miaolingian, the Mianchi area of North China is preserved as black clots within thrombolites and have dendritic and spherical outlines when viewed with a petrographic microscope. These remains, visible under scanning electron microscope (SEM), also comprise spherical or rectangle capsules. These capsules are made up from external envelopes and internal calcite with numerous pits, which closely resemble modern benthic coccoid cyanobacteria. These pits are between 2 μm and 4 μm in diameter and are interpreted here to represent the remnants of degraded coccoid cells, while the calcite that surrounds these pits is interpreted as calcified thin extracellular polymeric substances (EPS). In contrast, associated capsular envelopes represent thick EPS mineralized by calcium carbonate with an admixture of Al-Mg-Fe silicates. Dendritic ‘thalli’ are typically stacked apically because of the repeated growth and calcification of these capsules. Carbon and oxygen isotope results are interpreted to indicate that both photosynthesis and heterotrophic bacterial metabolism (especially sulfate reducing bacteria) contributed to carbonate precipitation by elevated alkalinity. *Epiphyton* are therefore here interpreted as colonies of calcified coccoid cyanobacteria, and the carbonate-oversaturated seawater during the Cambrian was conducive to their mineralization.

## Introduction

*Epiphyton* Bornemann (1886) [1] is one of the best known calcareous genera from Early Paleozoic shallow-marine limestones [2]. These dendritic microfossils have been widely reported in Early-to-Middle Cambrian [3–6] and Early Ordovician reef systems [7], are also seen sporadically in the Silurian [8], and then undergo a resurgence in the Late Devonian [9, 10] before disappearing from the fossil record in the Cretaceous [11]. The phylogenetic position of this genus also remains debated; *Epiphyton* was originally classified within the red algae [12, 13] and was placed within the Rhodophyta by Luchinina and Terleev [14] who compared Cambrian samples from Siberia with thalli samples from living *Corallina*. In earlier work, both Hofmann [15] and Poncet [16] had assumed that *Epiphyton* formed by repeated growth and the synsedimentary calcification of colonies of coccoid blue green algae, a view that was later also shared by Pratt [4]. It is noteworthy that *Epiphyton* branches always co-occur with *Renalcis* chambers, and that both of these genera have been shown to be the most common end-members of a series of salient, partly intergrading morphotypes [4]. Luchinina considered other calcimicrobes similar to *Epiphyton* to be growth stages within the life cycle of this genus, including *Renalcis*, *Izhella*, *Chabakovia*, *Shuguria*, *Gemma*, and the dendroid form *Korilophyton* [14].

Irrespective of these debates, the genus *Epiphyton* does appear to be the first widespread fossil group characterized by the fact that both its cells and colonies are preserved by some form of either calcification or calcite secretion [17]; some researchers have attempted to utilize these traits to probe the cellularity of this genus. Korde, for example, recognized the presence of well-developed cellular microstructure in the branches and septa of this genus, and interpreted cells attached to swellings and clots as sporangial reproductive structures [3, 12]. Brasier later noted that some chambers of the small and silicified *Renalcis* comprise a chain of spherical cells ~2 μm in diameter [18]. All of these so-called cellular structures, however, remain controversial; although the calcification of *Epiphyton* has been discussed for many years, even the body structure of this genus remains unclear. It is therefore essential to scrutinize the possible cells of this genus in more detail and to determine whether, or not, intracellular structure can be recognized. Thrombolites from the Cambrian Stage 5 Mianchi area of the North China platform enable an entirely new angle on ultramicroscopic studies on *Epiphyton*. The aim of this paper is to identify both *Epiphyton* cells and their appendages based on observations and to hypothesize the classification of this genus. Further, by determining the occurrence of both microbe-induced mineralization and chemical precipitation, and interpreting the process of calcification, it has at last been possible to establish a model for this dendritic genus.

## Geological setting

**Fig 1:**
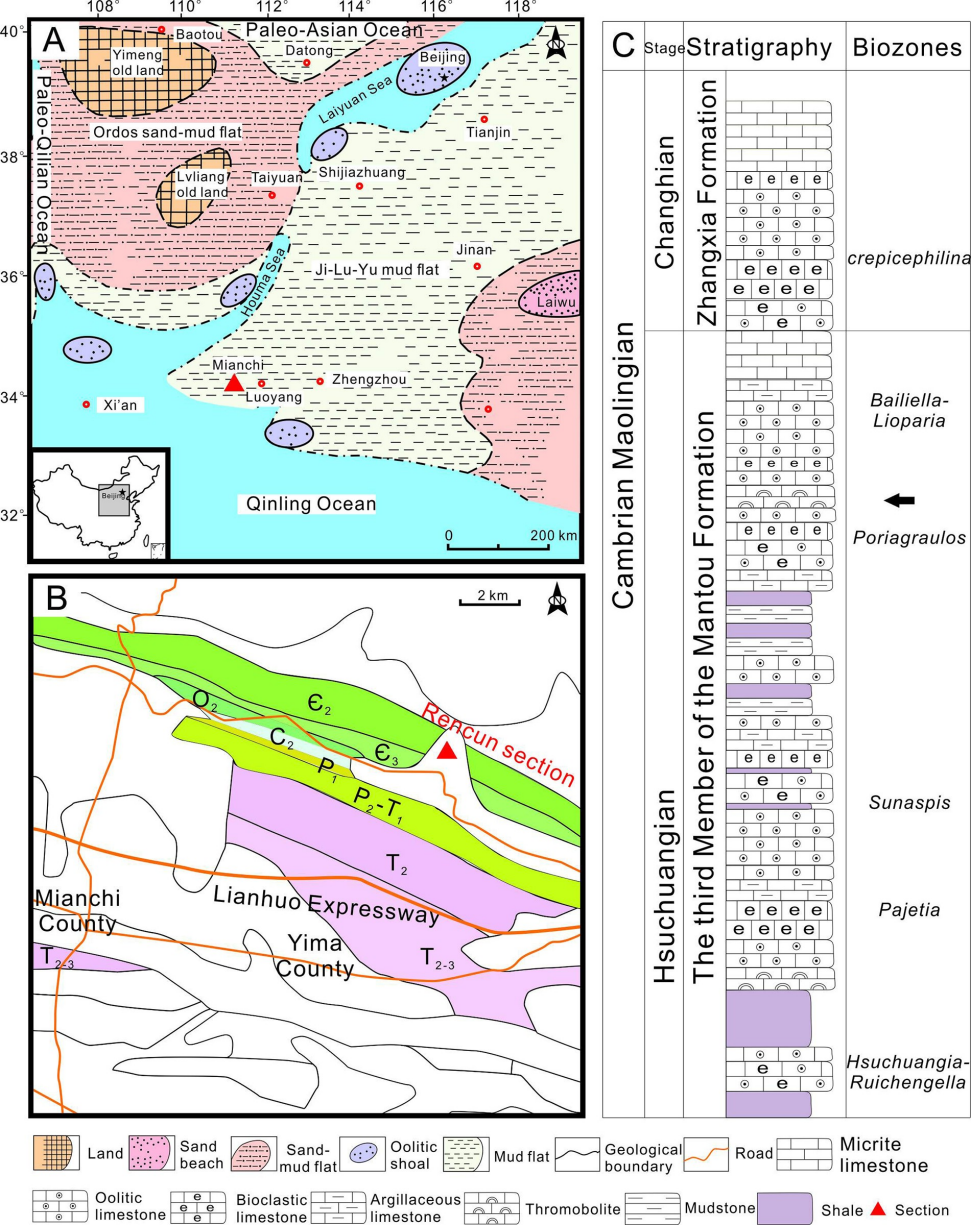
The geological setting and stratigraphy of the study area. (A) Paleogeography of the North China Platform in the early period of Cambrian Epoch 3 (modified after Feng [21]). (B) Simplified geological map of the Mianchi area. The white area corresponds with the Quaternary. (C) Stratigraphic column of M_3_ Fm. in the Rencun section. The black arrow indicates fossil beds.

The North China Platform was located near the paleoequator during the Cambrian [19] and comprised a stable epeiric sea that was surrounded by abyssal troughs (e.g., the northern Paleo-Asian Ocean, the Southern Qinling Ocean, and the western Paleo-Qilian Ocean). Deposition of the whole of this block began during Cambrian Epoch 2 in the aftermath of widespread transgression, subsequent to a long period of weathering since *ca*. 850 Ma [20]. During the early Hsuchuangian within Cambrian Miaolingian, enormous tidal flats covered the whole of this platform, ranging from sand-mud areas in the western Ordos Basin to mud flats in the eastern Ji-Lu-Yu area, separated by the Laiyuan and Houma seas (Fig 1A). A carbonate platform was gradually established via sustained transgression from the southwest, and by Changhian time this was dominated by ooid shoals [21].

**Fig 2:**
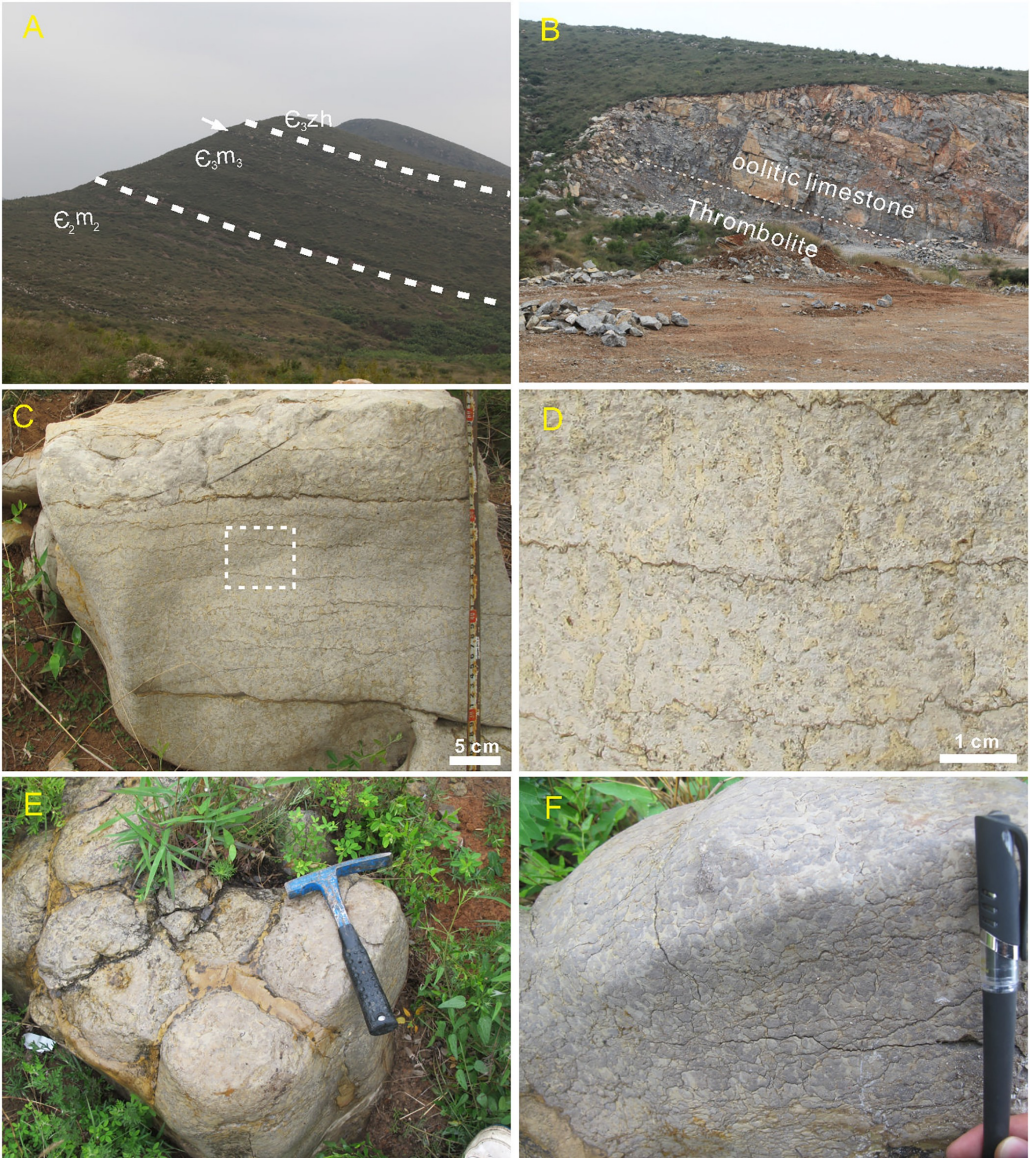
Photograph of section and thrombolites. (A) Overview of the Cambrian succession in the study area. The white arrow denotes the thrombolite horizon (B) Sharp contact between microbialite and overlying oolitic limestone (dashed line). (C) Stratiform thrombolite comprising yellow and black clots. (D) Enlargement of the rectangular area in (C) to show bifurcating clots perpendicular to bedding surfaces and truncated by the upper argillaceous stylolite. (E) Thrombolites comprise domed mounds and argillaceous interstitial matter. Hammer length = 33 cm. (F) Domed mounds showing the grey infillings and black clots that stack perpendicularly to bedding. Gel pen length = 14 cm.

The Mianchi area is located on the western Ji-Lu-Yu mud flat adjacent to the Qinling Ocean coastline (Fig 1A). The Rencun section (Fig 1B) within this region lies to the northeast of Mianchi County and is characterized by continuous and well-exposed Paleozoic units. The Cambrian in this region comprises (in ascending order) the Xinji and Zhushadong Formations of Series 2, followed by the Mantou and Zhangxia Formations of Miaolingian. The third member of the Mantou Formation (M_3_ Fm.) mainly comprises purplish red and greenish yellow shales interbedded with glauconitic sandstones, siltstones, and bioclastic limestones at their base, a purplish red shale interbedded with mud-striped and oolitic limestones in the middle section, followed by a dark grey and thick layer of mud-striped and oolitic limestones (Fig 1C). The presence of widespread oncolites are used to mark the lithostratigraphic boundary within this section with the overlying Zhangxia Formation [22], and five trilobite zones (Fig 1C) have also been identified, including *Hsuchuangia*-*Ruichengella*, *Pajetia*, *Sunaspis*, *Poriagraulos*, and *Bailiella-Lioparia* [23], which are also indicative of Cambrian Miaolingian [24]. *Epiphyton*-bearing thrombolites also occur within this section, are ~3 m in thickness, located in the upper part of M_3_ Fm. (Fig 2A), and are overlain with a thickly-bedded oolitic limestone (Fig 2B). The whole of this study interval is indicative of a shallow subtidal sedimentary environment.

## Materials and methods

A total of 24 samples were collected for this study from each thrombolite unit. Each sample was then processed into at least eight thin sections, one polished slab and one rock platelets, before petrographic (ZEISS Axioskop 40 Pol, Germany) and stereoscopic microscopes (ZEISS SteREO Discovery V20, Germany) were utilized to observe the optical characteristics of *Epiphyton*. Rock platelets were etched with 5% formic acid for 5 s before being rinsed with distilled water. This etching method for the identification of cyanobacterial sheath and capsule remains has been utilized in previous studies on both modern and ancient calcified cyanobacterial mats [25–27]. Splintered samples were then sputtered with gold to enhance conductivity, and were examined using a scanning electron microscope (SEM; FEI quanta 250, USA) operating at 10–15 kV before an accessorial Energy Dispersive Spectrometer (EDS; Bruker, Germany) was applied for multi-element determinations. All micro-level examinations were carried out in the Key Laboratory of Biogenic Trace and Sedimentary Minerals, Henan Polytechnic University, Jiaozuo, China. All thrombolite samples and thin sections are publicly deposited in the ichnofossil showroom of Henan Polytechnic University, Jiaozuo, China under catalogue numbers PZ160612–PZ160641, and all of them are accessible by others. No permits were required for the described study, which complied with all relevant regulations.

Carbon (δ^13^C_carb_) and oxygen (δ^18^O_carb_) carbonate isotopes were analyzed at the State Key Laboratory of Palaeobiology and Stratigraphy of the Nanjing Institute of Geology and Palaeontology, Chinese Academy of Sciences. To do this, ten micro-zone samples from *Epiphyton*-bearing black clot and grey filling nearby were obtained using a dental drill, and an aliquot of 80–100 μg of sample powder was reacted with orthophosphoric acid for 150–200 s at 72 °C in a Kiel IV carbonate device. The CO_2_ gas released was then tested for δ^13^C and δ^18^O with a MAT-253 mass spectrometer; isotope values are reported relative to the V-PDB standard. The analytical precision based on duplicate analyses is better than ± 0.04‰ for δ^13^C and ± 0.08‰ for δ^18^O.

## Results

Two thrombolite forms were recognized in the outcrop. The first of these, khaki-colour stratiform thrombolites (Fig 2C), are up to 40 cm in thickness and comprise multiple yellow and black clots. These bifurcating clots (like branches) are less than 5 cm in height and approximately equal in diameter, while branches run perpendicular to bedding surfaces and are truncated by the argillaceous stylolite above (Fig 2D). The second kind of thrombolite seen in this section comprises a series of domed mounds and argillaceous interstitial material (Fig 2E); each of these mounds is 20–40 cm in diameter and 60–100 cm in height, comprised of black clots and grayish yellow fillings (Fig 2F). These clots occupy up to 70% of the available space and are stacked vertical to bedding (Fig 2F).

**Fig 3:**
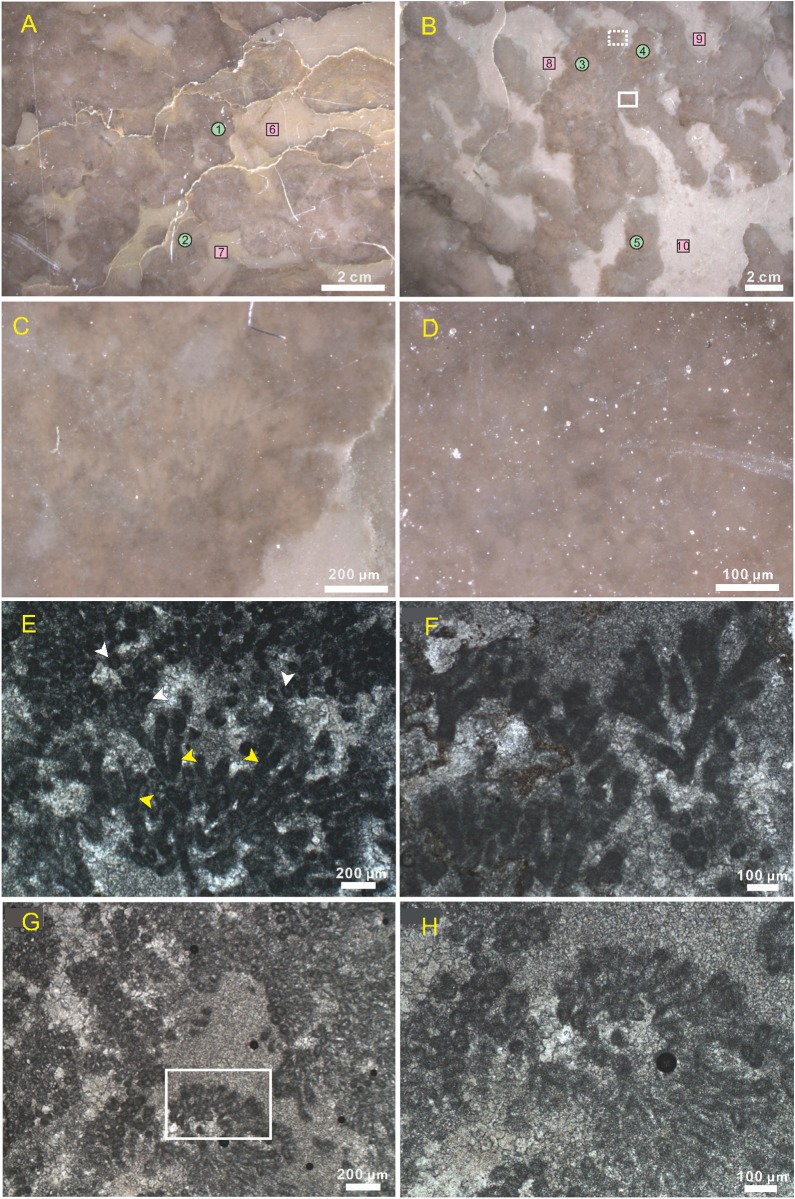
Photomicrographs of clot and calcified cyanobacteria. (A), (B), (C), and (D) show polished blocks of thrombolites viewed under reflected light, while (E), (F), (G) and (H) show calcified cyanobacteria viewed under transmitted light. (A) Clear boundary between a black clot and grey infilling. The positions of the figure represent geochemical sampling points that are listed in Table 1. (B) Brown patches in the dendritic clot suggest non-homogeneous internal components. (C) Enlargement of the rectangular (dashed line) area in (B) showing brown dendritic branches. (D) Enlargement of the rectangular (full line) area in (B) showing spherical colonies. (E) The fine calcite in branches (yellow arrows) and spheres (white arrows) has darker outlines compared to peripheral coarse calcite. Co-occurrence of spheres and branches may indicate their affinity. (F) Dichotomous branches of *Epiphyton*. (G) *Tubomorphophyton* composed of thin micritic walls and filled with microsparite. (H) Close up of the rectangle in (G) showing tubiform structure.

Polished thrombolite sections clearly show that there is a sharp contrast between clots and fillings in both colour and components (Fig 3A and 3B). These clots are dark coloured and have clear boundaries and sometime stylolites can form these edges due to different physical properties on both sides (Fig 3A). Similarly, the grey fillings are also characterized by their uniform color and constituents. Further observations revealed that interior clots can be separated into two distinct regions, brown patch and a black background (Fig 3B). These brown areas comprise two communities, dendritic (Fig 3C) and capsular colonies (Fig 3D); the first of these consists of between three and five branches that have the same diameters, up to 400 μm in length (Fig 3C), while spherical aggregates are scattered about and are also equal in size, and 30–50 μm in diameter (Fig 3D).

Additional disparities are seen in both morphology and composition when samples are viewed with a petrographic microscope. Observations show that branches and spherical aggregates are comprised of fine-grained minerals, which has a darker outline under transmitted light (Fig 3E). Each dendritic clump initially comprises just one trunk, and gradually branches dichotomously upwards (Fig 3E and 3F). Further, additional tubiform microbes (*Tubomorphophyton*) with branches and spheres that also have thin micritic walls and internal microsparite (Fig 3G and 3H) are also found within the same horizons, leading to the possibility that both structures represent the same type of organism with a different form of preservation. Further ultrastructural studies will be required to resolve these issues.

**Fig 4:**
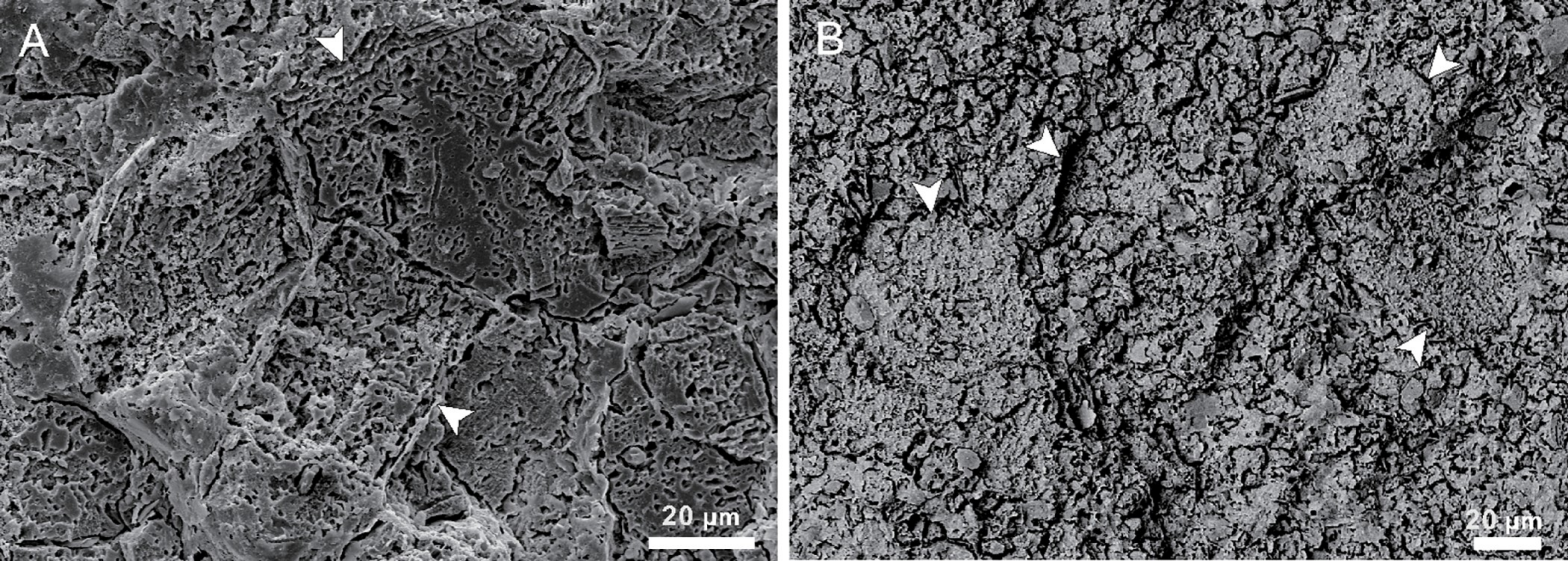
Scanning electron microscope images of *Epiphyton* within Cambrian thrombolites. (A) Regular rectangles (white arrow). (B) Spheres (white arrow) with fine grained calcite and external coatings, back scattered electron mode.

**Fig 5:**
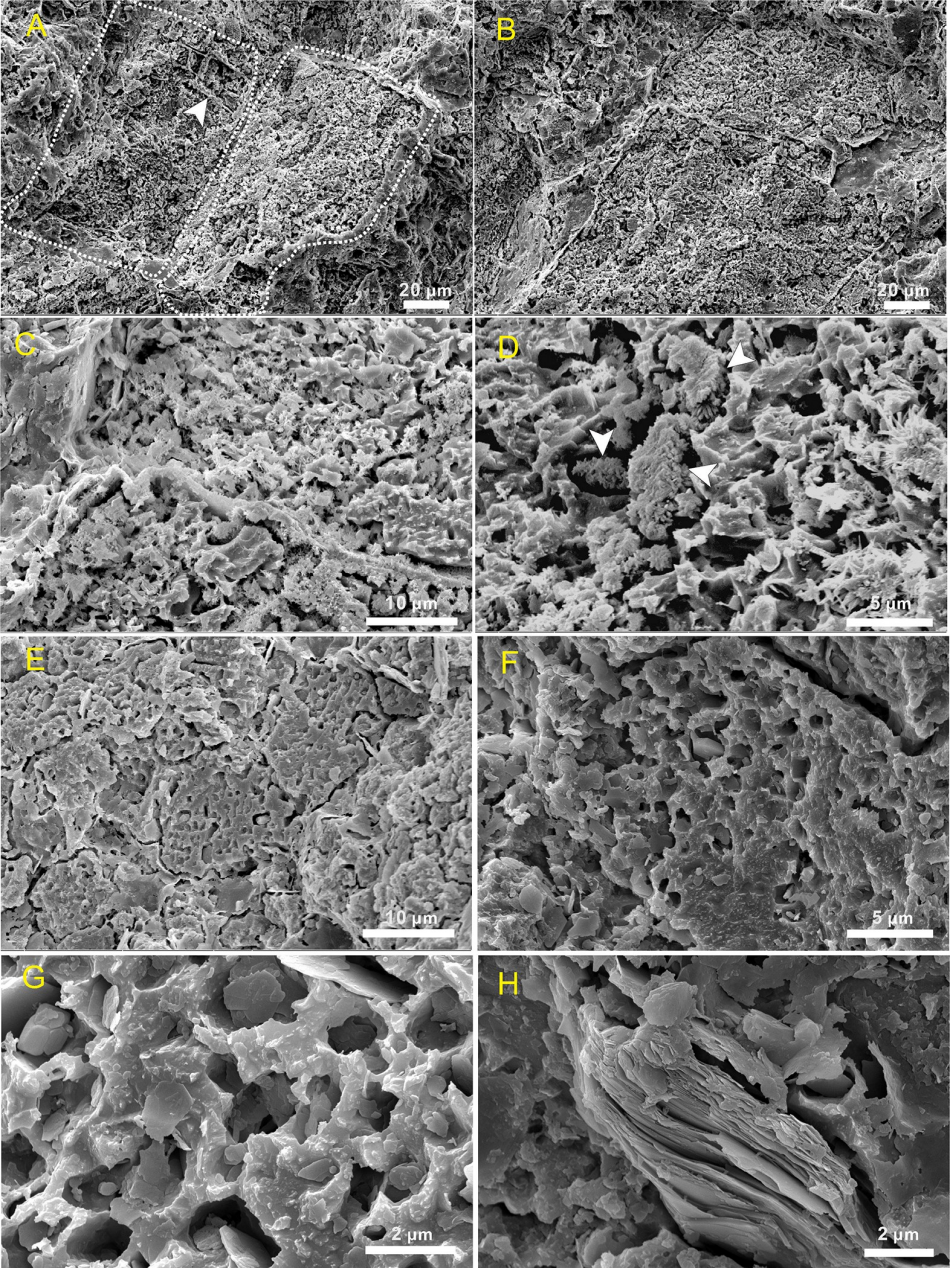
Scanning electron microscope images of rectangular and spherical capsules from Cambrian thrombolites. (A) Rectangular capsules (marked with dotted line) and smaller rectangles (marked with arrow). (B) Two rectangles connected end-to-end and sharing the same envelope. These capsules comprise micritic calcite in contrast to the surrounding sparry calcite. (C) Close-up view of envelopes that separate two capsules. (D) Some acicular or fibrous clay minerals in pits, marked with white arrows. (E) Irregular spherical capsules. (F) Close up of capsule showing pits arrayed equidistantly within calcite. (G) Hollow and round pits containing scattered lamellar minerals. (H) Lamellar envelopes around the capsule.

Two ultra-structures are revealed by SEM, regular rectangles (Fig 4A) and irregular spheres (Fig 4B). The rectangles observed are 50−100 μm in width and more than 100 μm in length. Each one has a thin external envelope that separates the inner fine-grained calcite from the exterior coarse form (Fig 5A and 5B); these envelopes, 1−2 μm in thickness, can also be found internally, separating many smaller rectangular areas (Fig 5A). These rectangles connect with each other end-to-end and share the same envelopes (Fig 5B and 5C); these inner minerals are characterized by massive pits (2 μm average diameter) that are filled with some acicular or fibrous clay mineral (Fig 5D). The irregular spheres encompass a wide range of sizes, 10–80 μm in diameter (Fig 5E), and each comprises an exterior capsular envelope and inner pit-enriched minerals. These envelopes also usually drop off, leaving an interspace, while these round pits are average 1–2 μm in diameter, arranged equidistant from one another within the same spheres (Fig 5F) but variable in others. At the same time, the number of pits varies; most are hollow, but some are filled with lamellar minerals (Fig 5G) that derive from the exterior envelopes, themselves up to 5 μm thick (Fig 5H). We define the rectangular and spherical structures with external envelops into capsules.

**Fig 6:**
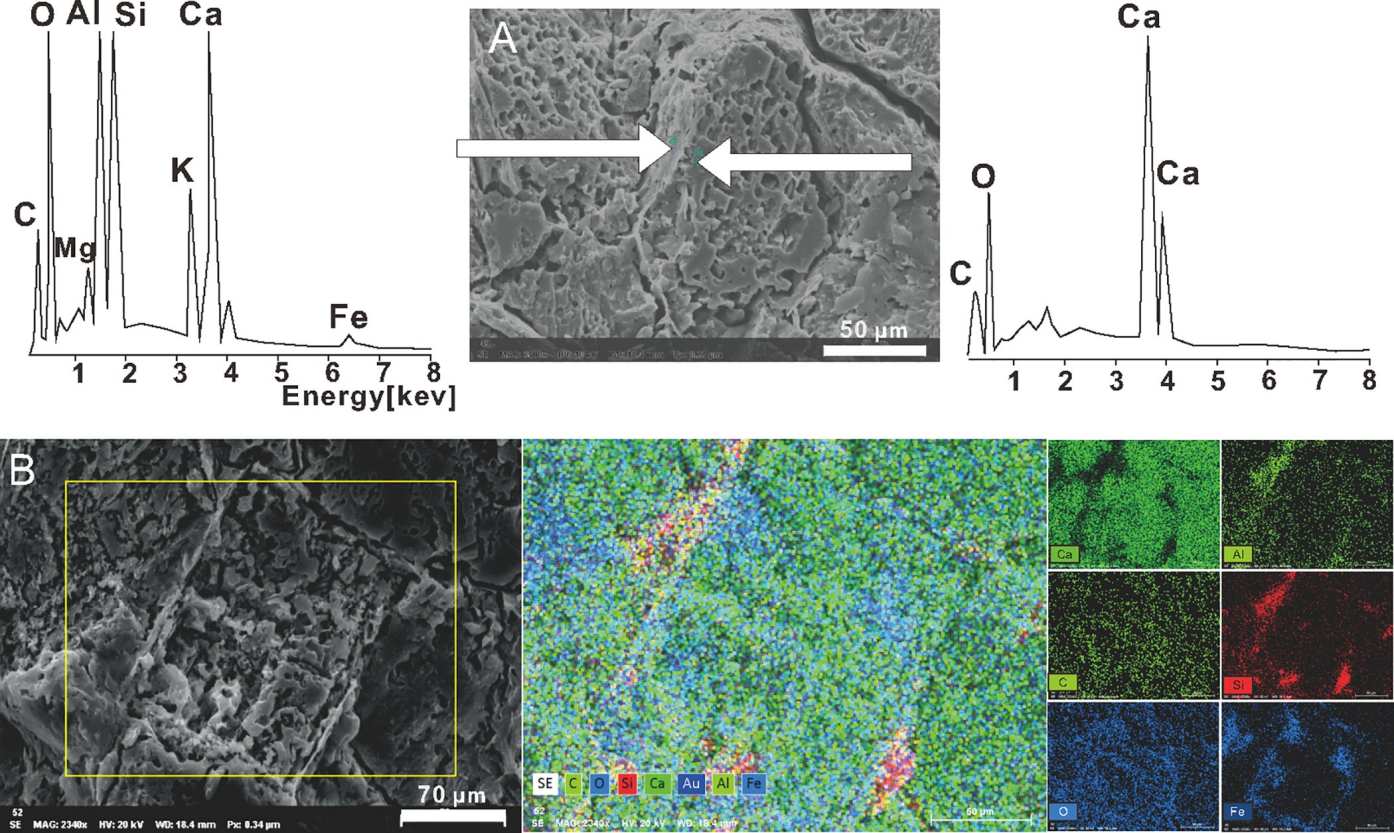
**EDS spectra of mineralized spherical capsule (A, spot analyses) and rectangular capsule (B, panel analyses).** Scale bars: A– 50 μm; B– 70 μm.

Analyses using EDS demonstrate that the elemental composition of the two different forms are identical (Fig 6A and 6B). Envelopes are comprised of Ca, Si, Al, Fe, K, Mg, O, and C, and therefore are calcium carbonate containing large admixture volumes of silicates (Fig 6A). In contrast, the encapsulated material comprises Ca, C, and O, and so is definitely a pure calcium carbonate (Fig 6B). The results therefore show that the structures are composed of a laminar silicate envelope and encapsulated pit-enriched calcite.

The C and O isotope composition of the black clots and grayish infillings are both characterized by slightly negative δ^13^C and markedly negative δ^18^O values (Table 1). Analyses reveal that the C isotope composition of the black clots varies between −0.348‰ and −0.559‰, more negative than is the case for the greyish infillings which range between −0.181‰ and −0.515‰. In contrast, O isotope values for these black clots range between −7.562‰ and −7.874‰, more negative than the greyish fillings which range between −6.640‰ and −7.583‰. Most isotopic results are very similar to benthic coccoid cyanobacterial calcified mats from Late Jurassic open marine sediments in central Poland [28] (Table 1), except for carbon isotopic trend that are opposite in coarse grained calcite (corresponding to black clots) and fine grained calcite (corresponding to greyish infillings).

**Table 1:** δ^13^C and δ^18^O for black clots (B) and greyish fillings (G) of the Cambrian thrombolites (Sampling points are shown in Fig 3A and 3B), and the Late Jurassic calcified counterparts (shadow).

	Greyish fillings	δ^13^C_carb_(‰, PDB)	δ^18^O(‰)	Black clots	δ^13^C_carb_ (‰, PDB)	δ^18^O(‰)
Cambrian **samples** in this paper	G6	-0.204	-7.439	B1	-0.348	-7.645
	G7	-0.207	-7.243	B2	-0.559	-7.562
	G8	-0.387	-7.583	B3	-0.341	-7.679
	G9	-0.181	-6.640	B4	-0.373	-7.874
	G10	-0.515	-7.543	B5	-0.442	-7.742
	G, n = 5	−0.299 ± 0.04	-7.290 ± 0.08	B, n = 5	−0.413 ± 0.04	-7.700 ± 0.08
Jurassic counterparts	Fine grained calcite	-0.15	-7.07	Coarse grained calcite	0.15–0.34	-7.81–-7.89

## Discussion

### The cellularity of *Epiphyton*

The morphological features in calcimicrobes of this study are all indicative of *Epiphyton* Bornemann (1886), and the spheres and branches may be the remains of calcified extracellular polymeric substances (EPS). Although the majority of previous researchers have noted the co-occurrence of thalli (branches) and capsules when studying *Epiphyton*, individuals have nevertheless held totally different opinions. Pratt, for example, regarded these spheres as just *Renalcis* chambers co-occurring with *Epiphyton* [4], while Luchinina more boldly hypothesized that the former genus (capsules) might be an instar of the latter (thallus) [14]. In addition, other researchers have formed the idea that *Renalcis*-resembling structures might be a diagenetic stage of chambered *Epiphyton* [4, 29, and 30]. The evidence for the hypotheses seem persuasive in this case: the transformation from upright thalli to spherical form (dashed rectangle in Fig 3E); the similarity in weak optical heterogeneity between micritic walls and inner cavity (Fig 3F). All evidences trigger a new round of question, whether two forms are kindred?

**Fig 7:**
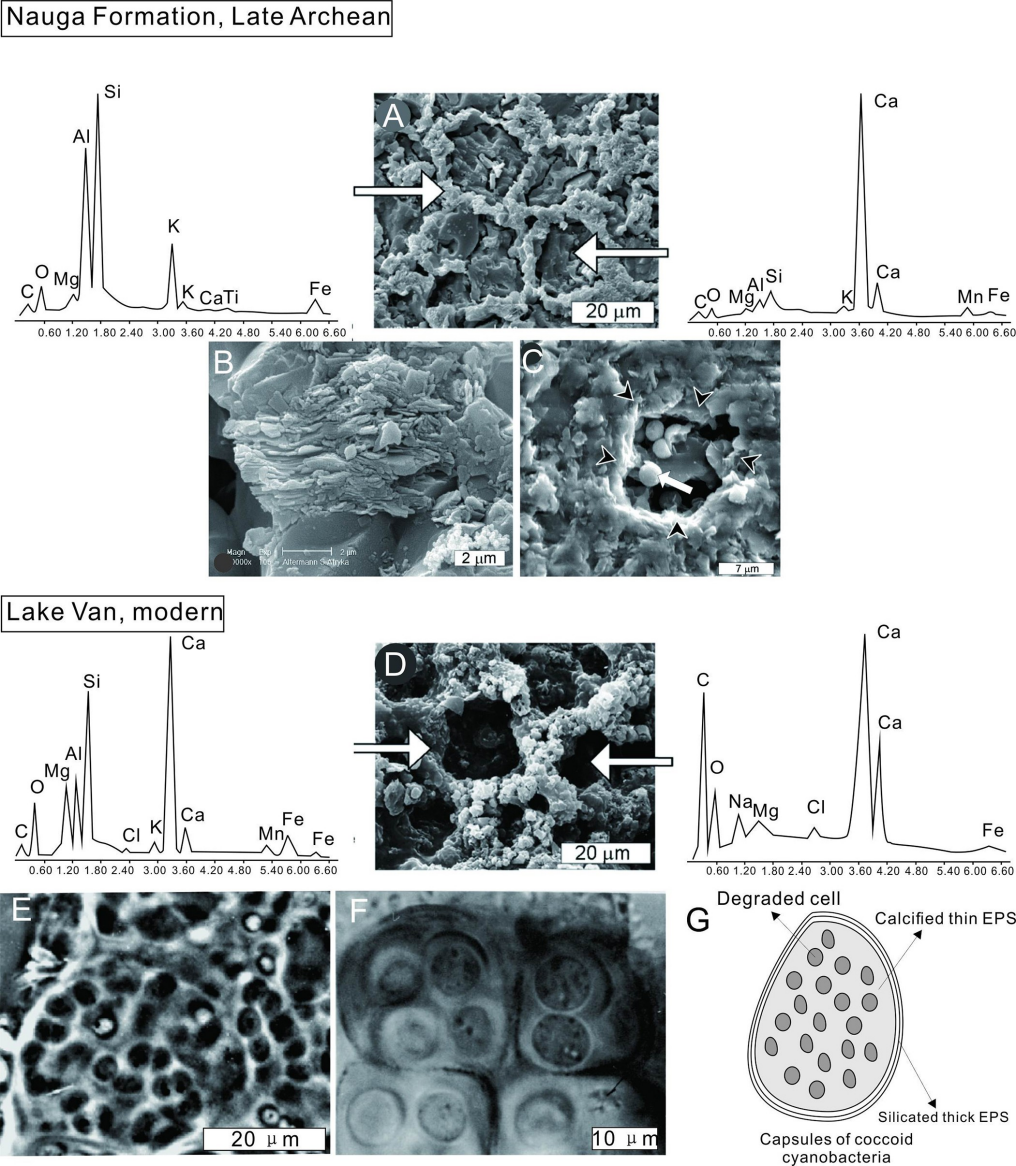
Archean and modern analogues of *Epiphyton*. (A–C) Archean calcified coccoid cyanobacteria (modified after Kazmierczak *et al*. [26]) showing honeycomb patterns, layered envelopes, pits with silicified cocci and identical elements. (D) A modern coccoid cyanobacterial mat from Lake Van (Turkey) (modified after Kazmierczak *et al*. [26]) showing honeycomb structure and similar elements. (E) Examples of degradation cocci and remaining pits from Lake Van [26]. (F) Colonies with a number of cells contained within an outer membrane, which represent a prototype of mineralized capsules [39]. (G) Model to show the correspondence between cocci colonies *in vivo* and the mineralized capsule.

Comparing these microstructures with their counterparts, provides an effective way to build correspondence between cells *in vivo* and their mineralized remnants. Possible analogues for these ancient Chinese structures are calcified microbial mats composed of coccoid cyanobacteria from modern mats from Lake Van (Turkey) [31–33], which are similar also to microbia in the Neoarchean Nauga Formation (South Africa) [26]. The cobweb-like etching patterns seen in the Neoarchean Nauga Formation (Fig 7A) also correspond with mineralized microbial (cyanobacterial) mats and exhibit a great degree of similarity with *Epiphyton* (Fig 6E). EDS analyses indicate that the walls of these South Africa structures comprise Ca, Si, Al, Fe, K, Mg, O, and C, while their inner minerals are pure calcium carbonate (Fig 7A). Layered envelopes (phyllosilicates) (Fig 7B) and pits (Fig 7C) have also been described from this sequence, although there is nevertheless quite a large difference between the two; pits in Neoarchean counterparts contain silicified spheres 2–4 μm in diameter instead of vacancies and are regarded as cocci. Similar spheres have also been reported in Late Devonian limestones from the Holy Cross Mountains [27], and the Lake Van microbialite modern analogues exhibit the same web-like features and have a similar chemical composition (Fig 7D) with the exception of the soluble elements K, Na, and Cl which readily run off during the diagenesis [26, 27]. The cocci in these specimens tend to be < 10 μm in diameter and belong to the order Chroococcales, either as members of the family Entophysalidaceae (exemplified by the genera *Entophysalis*, *Cyanosarcina*, *Pseudocapsa*, *Paracapsa*, *Lithocapsa*, and *Chlorogloea*), or within the order Pleurocapsales (exemplified by the genera *Pleurocapsa*, *Chroococcidiopsis*, *Xenococcus*, and *Chroococcopsis*) [34, 35]. When cyanobacteria die, the organic molecules and entire cellular structures, together with their membranes, collapse and shrink very rapidly under the lytic action of enzymes (Fig 7E), but some highly chemically resistant molecules exist, such as EPS [36, 37]. Besides, some biominerals are induced by microbes in EPS, which are more resilient than organic bacterial structures [38]. Therefore, these mucilaginous sheaths have a higher preservation potentiality than contemporaneous cellular structures, leaving pits in the fossil record. Due to cell division, the innermost envelopes represent the last EPS produced by the cell prior to death and conform best with cellular shape, while outermost ones enclosing all cell aggregates may be the prototypes of mineralized capsules (Fig 7F). At the same time, research on the Holocene species *Entophysalis major* from stromatolites at Shark Bay confirm that the tight packaging of cells and envelopes could cause polyhedral deformation and develop into cubically flattened capsules [15]. These powerfully supported rectangular capsules are homologous with spherical ones, and both are coccoid cyanobacteria aggregates.

Observations corroborate the correspondence between colonies of coccoid cyanobacteria and mineralized *Epiphyton* (Fig 7G), specifically 1): *Epiphyton* comprise coccoid cyanobacteria; 2) Pits are probably the remains of degraded cells; 3) Encapsulated calcium carbonate corresponds with EPS that adjacent to individual cells, and; 4) The silicate capsule envelopes correspond with the outermost and thickest EPS.

### Bioinduced mineralization within *Epiphyton*

Cyanobacteria are capable of triggering precipitation via biologically-induced mineralization by elevating alkaline levels in their micro-environment, closely related to metabolic activities [40, 41]. However, the specific mechanisms that underlie this calcification still remain unclear and controversial [42]. Two calcification processes have been studied extensively to date, including cyanobacterial photosynthesis and the sulfate reduction by sulfate reducing bacteria (SRB).

**Fig 8:**
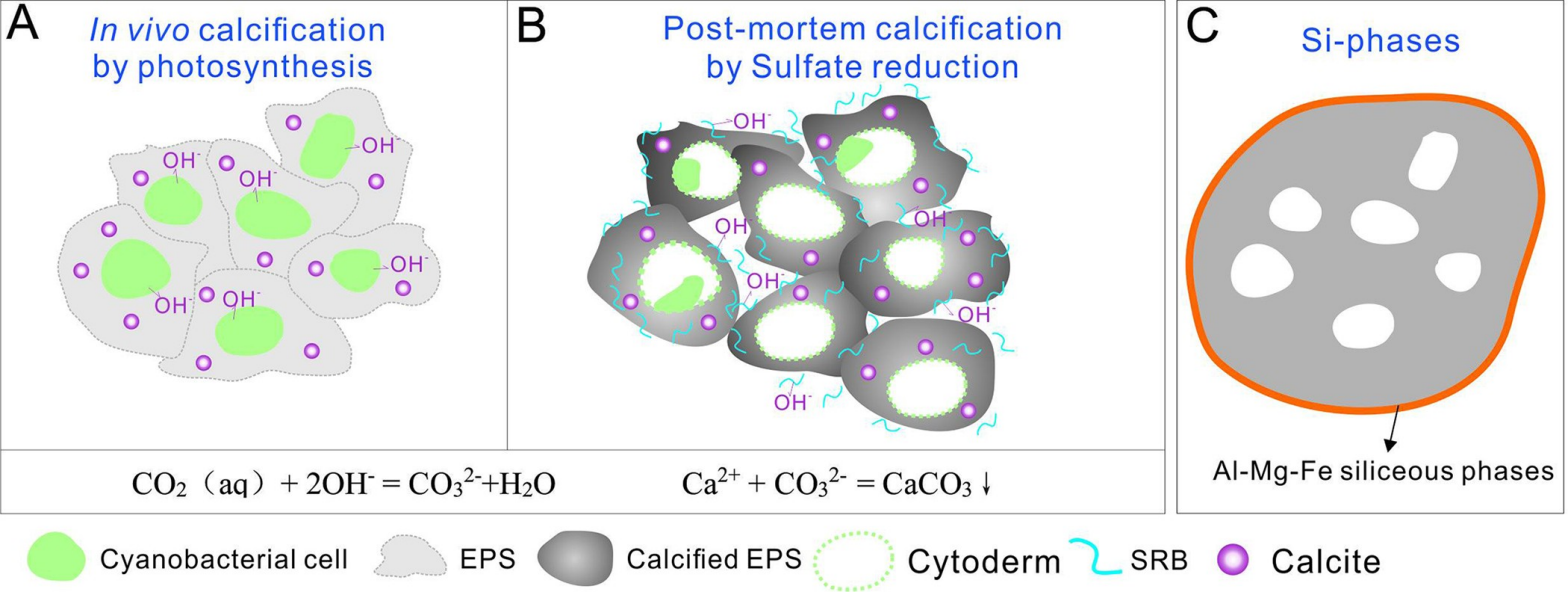
Capsule mineralization. (A) *In vivo* calcification for elevated alkalinity via photosynthesis. (B) Post-mortem calcification via sulfate reduction which leads to the alkalinity produced by the metabolism of sulfate reducing bacteria. Chemical equation showing how rising alkalinity contributes to precipitation. (C) Authigenic Al-Mg-Fe silicate minerals that encase the capsule.

It is noteworthy that although Cambrian samples have undergone diagenetic alteration (δ^18^O values are less than -5al, the more negative δ^18^O values of the black clots (mean -7.700‰) compared to greyish infills (mean: -7.290‰) supports the hypothesis that calcification is related to photosynthesis as cyanobacteria preferentially utilize H^13^C^18^O^18^O^18^O^-^ in photo assimilation, resulting in the relative enrichment of ^16^O within the mat [43]. Thus, during oxygenic photosynthesis, this CO_2_-concentrating mechanism can elevate pH values, which increases the saturation state to calcium carbonate [42]. This means that just a continuous alkaline supplement can sustain precipitation within living cyanobacterial cells. Specific mineralization processes therefore proceed as the cyanobacterial photosynthesis accumulates a massive volume of OH^-^ ions around the EPS, and these then react with dissolved CO_2_ react to produce CO_3_^2−^ (Fig 8A). In addition, a large amount of cations, such as Ca^2+^ and Mg^2+^, are adsorbed by negatively charged EPS; thus, once Ca^2+^ are released (EPS degrades under the impact of cytolysis), precipitation immediately occurs within the EPS and its properties influence the mineralogy of precipitated CaCO_3_ crystals [44].

Sulfate reduction is considered as a major process in microbialites [45]. Modern experimental observations show that when O-dependent metabolism is suspended, for example during the night, anaerobic heterotrophy, especially sulfate reduction, begin to perform import role to maintain alkalinity [41]. Photosynthesizing communities tend to preferentially fix ^12^C, and this means that biomass typically over 20‰ is depleted in ^13^C relative to the marine inorganic C reservoir [46]. This viewpoint would therefore predict that the bioinduced calcite in black clots should be rich in ^13^C; isotope analyses indicate, however, that the δ^13^C values of these regions (mean −0.413‰) are relatively depleted compared with gray fillings (mean −0.299‰), indicating the involvement of SRB because δ^13^C value of carbonates precipitated by sulfate reduction can range up to between −20‰ and −21‰ even though some less depleted cases still exist [47]. At the same time coccoid cyanobacteria EPS separates numerous heterogeneous micro-domains, which support SRB metabolism by serving as both an energy and carbon source [45]. The metabolism of SRB can therefore generate both OH^−^ and even CO_3_^2−^, as follows [48]:

$$[(CH_{2}O{)}_{106}(NH_{3}{)}_{16}(H_{3}{PO}_{4})]+53SO_{4}^{2-}\to 106CO_{2}+16NH_{3}+53S^{2-}+H_{3}{PO}_{4}+106H_{2}O;$$ (1)

$$NH_{3}+CO_{2}+H_{2}O\to 2NH_{4}^{+}+CO_{3}^{2-},$$ (2)

and;

$$NH_{3}+H_{2}O\to NH_{4}^{+}+OH^{-}.$$ (3)

Thus, post-mortem mineralization is associated with SRB activities within dead cyanobacterial biomass, and SRB cytolysin can also degrade cyanobacterial EPS and release Ca^2+^ and Mg^2+^ cations which rapidly precipitate with ambient CO_3_^2-^ [49–51] (Fig 8B).

The microorganisms induced precipitation of the silicate phase have been the subject of extensive studies [52–56]. Tazaki argued that coccidial bacteria could promote nucleation of silicates on coccoidal bacteria cell walls by immobilization of metal ions [52]. The observations from modern calcareous microbialites in Van Gölü (Lake Van) confirmed the formation process of siliceous mineral phases [56]: abundant minute aragonite grains precipitated first *in vivo* in the EPS; then these grains were rapidly succeeded and/or supplemented in the dead biomass of the cyanobacterial mat by authigenic Al-Mg-Fe siliceous phases. Fein et al. concluded that Si-phase mineralization is an effect of Fe and Al adsorption [57] and that bacterial EPS may serve as templates for Fe/Al oxide precipitations [52]. These metallic cations react with aqueous SiO_2_ available in the medium. In this way, the formation of silica and Al-Si phases begins [57]. These hydrous precursors dehydrate over time and are converted to capsule envelopes (Fig 8C). These densified coatings seal up the inner cavity and protect the pit remains from diagenetic change. Authigenic siliceous phases significantly enhancing the fossilization potential of the mat-forming cyanobacteria [56].

### *Epiphyton* assemblages

**Fig 9:**
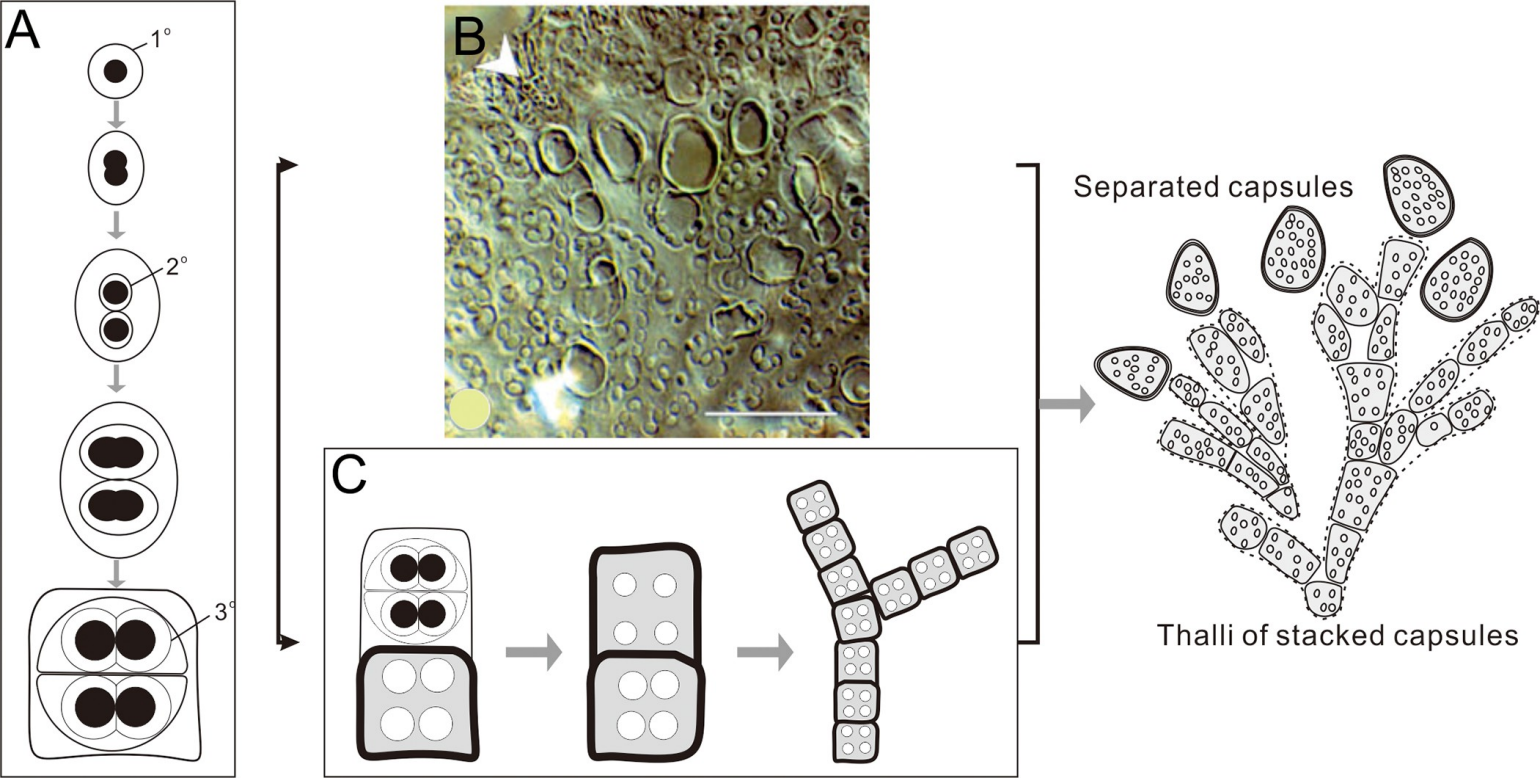
The cell division and assemble model for *Epiphyton*. (A) Cell division pattern showing two new daughter cells and gelatinous envelopes that are reproduced (modified after Golubic and Hofmann [39]). Envelopes in sequence are marked as 1°, 2°, and 3°. (B) A slightly calcified colony of modern coccoidal cyanobacteria (*Entophysalis* sp.) from Lake Van (Turkey) [27] showing how thicker mucilage envelopes stack vertically to be branches. Scale bars: 50 μm. (C) An apical growth model for *Epiphyton* implying that dendritic thalli formed by successive growth and the calcification of similar-sized colonies.

Coccoid cyanobacteria typically reproduce asexually, especially via binary fission. This attribute was first noted by Golubic and Hofmann who suggested that the presence of Precambrian Entophysalidaceae in stromatolites might have initially propagated via binary fission in much the same way as their Holocene counterpart *Entophysalis major*: the parent cell direct divides into two identical descendants during proliferation; the initial EPS around second-generation cells which are themselves enclosed by parental sheaths, and so on [39] (Fig 9A). In comparison, the cocci within *Epiphyton* exhibit the same patterns as calcified EPS over different generations presented in one capsule. Although at attempt was made in this study to count the number of pits to demonstrate whether, or not, this theory is appropriate, remolding by diagenesis to a greater or less extent seems to obscure the truth.

How do these coccoid cyanobacteria or colonies (capsules) construct their dendritic branches? Two conceivable models have been proposed to explain this, the degraded and calcified model of Kazmierczak *et al*. [26, 27] and the apical growth pattern model proposed by Pratt [4].

The degraded and calcified model seems to coincide very well with the situation seen in fossils without the requirement of a biological control. One fragment from a degraded and weakly calcified colony of the modern colonial coccoidal cyanobacteria (*Entophysalis* sp.) from Lake Van (Turkey) confirms that thicker mineralized mucilage envelopes (the glycocalyx) automatically pile up vertically during diagenesis [26, 27] (Fig 9B). This observation suggests that *Epiphyton* branches might be assembled in this way, although it remains hard to explain why these mineralized envelopes are equally sized (Fig 5B) as well as the meaning of the dichotomous branching pattern.

In contrast, the apical growth model (Fig 9C) perfectly explains these assembled capsules. Dendritic *Epiphyton* are formed by successive growth and calcified colonies along the branch. This hypothesis indicates that all branches are controlled by growth hormones that stimulate apical growth, and so might be analogous to algae. Further research will be required to determine which of these aggregates are capable of this ability.

## Conclusion

This study presents evidence that *Epiphyton* was constructed by colonies of calcified coccoid cyanobacteria. In these cases, branches are stacked in the form of a string of end-to-end capsules regarded here as cyanobacterial thalli. Thus, each of these capsules comprises an outermost Al-Mg-Fe silicate envelope and inner calcite infillings which are constituted from mineralized thick EPS and calcified thin sheaths, respectively. Some pits within inner calcite in these cases have been dismissed as remnants of degraded cocci, while the metabolism of cyanobacterial photosynthesis and SRB sulfate reduction both contribute to *in vivo* calcification and post-mortem changes by elevating ambient alkaline levels to the supersaturated calcium carbonate point. Al-Mg-Fe silicate are the last stage of mineralization when metallic cations absorbed in the EPS react with aqueous SiO_2_ in the water. Thus, massively abundant occurrences of *Epiphyton* in the Cambrian reflect an excessive seawater carbonate saturation state which promoted bioinduced calcification, the first such act within the Phanerozoic [58].

*Epiphyton* was widespread during the Paleozoic, especially in the Cambrian; indeed, the distribution of coccoid cyanobacteria almost encompasses the whole evolutionary history of life. Although the presence of *Epiphyton* in the Cambrian may be regarded as a special kind of coccoid cyanobacterial colony, but whether, this branched fossil is related to specific coccus gene expression or environmental indexes (i.e., marine chemical conditions and *PCO*_*2*_) will require further research.

## Supporting information

S1 Dataset The location of sampling points and the corresponding δ^13^C and δ^18^O value. (XLSX)

## References

[1] Bornemann JG. Die Versteinerungen des cambrischen Schichtensystems der Insel Sardinien nebst vergleichenden Untersuchungen über analoge Vorkommnisse aus anderen Ländern 1. Nova Acta der Kaiserlichen Leopoldinisch-Carolinischen Deutschen Akademie der Naturforscher. 1886;51(1): 1–147.

[2] Săsăran E, Bucur II, Pleş G, Riding R. Late Jurassic Epiphyton-like cyanobacteria: indicators of long-term episodic variation in marine bioinduced microbial calcification? Palaeogeography Palaeoclimatology Palaeoecology. 2014;401(5): 122–131.

[3] Korde KB. Vodorosli kembriya yugo-vostoka Sibirskoy platformy. Academy of Sciences of the U.S.S.R. Publishing House. 1961. (in Russian).

[4] Pratt BR. *Epiphyton* and *Renalcis*–diagenetic microfossils from calcification of coccoid blue-green algae. Aapg Bulletin. 1984;54(3): 948–971.

[5] Riding R. Calcified algae and bacteria In: Zhuravlev A.Yu., Riding R. (Eds.), The Ecology of the Cambrian Radiation. Columbia University Press, New York; 2001 pp. 445–473.

[6] Gandin A, Debrenne F. Distribution of the archaeocyath-calcimicrobial bioconstructions on the Early Cambrian shelves. Palaeoworld. 2010;19: 222–241.

[7] Coniglio M, James NP. Calcified algae as sediment contributors to Early Paleozoic limestones: evidence from deep-water sediments of the Cow Head Group, western Newfoundland. Journal of Sedimentary Research. 1985;55: 746–754.

[8] Riding R, Soja CM. Silurian calcareous algae, cyanobacteria, and microproblematica from the Alexander Terrance, Alaska. Journal of Paleontology. 1993;67(5): 710–728.

[9] Wray JL. Upper Devonian calcareous algae from the canning basin, Western Australia. Colorado School of Mines. Geochem. 1967;38 (5):719–833.

[10] Shen JW, Yu CM, Bao HM. A late-Devonian (Famennian) *Renalcis*-*Epiphyton*, reef at Zhanjiang, Guilin, South China. Facies. 1997;37(1): 195–209.

[11] Barattolo F. Mesozoic and Cenozoic marine benthic calcareous algae with particular regard to Mesozoic Dasycladaleans–In: Riding R. (ed.): Calcareous Algae and Stromatolites. Springer-Verlag; 1990 pp. 504–540.

[12] Korde KB. Vodorosli kembriya (Cambrian algae). Academy of Sciences of the U.S.S. R., Transactions of the Palaeontological Institute; 1973. (in Russian).

[13] Riding R, Toomey DF. The sedimentological role of *Epiphyton* and *Renalcis* in lower Ordovician mounds, southern Oklahoma. Journal of Paleontology. 1972;46(4): 509–519.

[14] Luchinina VA, Terleev AA. The morphology of the genus *Epiphyton* Bornemann. Geologia Croatica. 2008;61(2): 105–111.

[15] Hofmann HJ. Stratiform Precambrian stromatolites, Belcher Islands, Canada: relations between silicified microfossils and microstructure. American Journal of Science. 1975;275: 1121–1132.

[16] Poncet J. Hypothese relative a la morphogenese du thalle de *Renalcis* (Algue calcaire—Paleozoique) et affinite possible avec les Rivulariacees actuelles. Géobios. 1976;9(3): 345–351.

[17] Riding R, Voronova L. Affinity of the Cambrian alga Tubomorphophyton and its significance for the Epiphytaceae. Palaeontology. 1982;25: 869–878.

[18] Braser MD. Early Cambrian intergrowths of Archaeocyathids, *Renalcis*, and pseudostromatolites from South Australia. Palaeontology. 1976;19: 223–245.

[19] Yang Z, Otofuji YI, Sun Z, Huang B. Magnetostratigraphic constraints on the Gondwanan origin of North China: Cambrian/Ordovician boundary results. Geophysical Journal International. 2002;151: 1–10.

[20] Meng X, Ge M, Tucker ME. Sequence stratigraphy, sea-level changes and depositional systems in the Cambro-Ordovician of the North China carbonate platform. Sedimentary Geology. 1997;114(1): 189–222.

[21] Feng ZZ. Lithologic Paleogeography of Early Paleozoic of the North China Platform. Geological Publishing House, Beijing; 1990. (in Chinese with English abstract).

[22] Zhang W, Shi X, Jiang G, Tang D, Wang X. Mass-occurrence of oncoids at the Cambrian series 2–series 3 transition: implications for microbial resurgence following an early Cambrian extinction. Gondwana Research. 2015;28(1): 432–450.

[23] Liu YH. Cambrian and Ordovician of Henan province. Geological Publishing House, Beijing; 1991. (in Chinese with English abstract)

[24] Peng SC. The newly-developed Cambrian biostratigraphic succession and chronostratigraphic scheme for South China. Chinese Science Bulletin. 2009;54(22): 4161–4170.

[25] Kempe S, Kaźmierczak J. Satonda Crater Lake, Indonesia: Hydrogeochemistry and biocarbonates. Facies. 1993;28(1): 1–31.

[26] Kazmierczak J, Altermann W, Kremer B, Kempe S, Eriksson PG. Mass occurrence of benthic coccoid cyanobacteria and their role in the production of Neoarchean carbonates of South Africa. Precambrian Research. 2009;173(1): 79–92.

[27] Kazmierczak J, Kremer B, Racki G. Late Devonian marine anoxia challenged by benthic cyanobacterial mats. Geobiology. 2012;10(5): 371–83. 10.1111/j.1472-4669.2012.00339.x 22882315

[28] Kazmierczak J, Coleman MI, Gruszczynski M, Kempe S. Cyanobacterial key to the genesis of micritic and peloidal limestones in ancient seas. Acta Palaeontologica Polonica. 1996;41(4): 319–338.

[29] Woo J, Chough SK, Han Z. Chambers of *Epiphyton* thalli in microbial buildups, Zhangxia formation (Middle Cambrian), Shandong province, China. Palaios. 2008;23(2): 55–64.

[30] Adachi N, Nakai T, Ezaki Y, Liu J. Late Early Cambrian Archaeocyath reefs in Hubei Province, South China: modes of construction during their period of demise. Facies. 2014;60: 703–717.

[31] Kazmierczak J, Kempe S, Altermann W. Microbial origin of Precambrian carbonates: Lessons from modern analogues. The Precambrian Earth: Tempos and Events. 2004: 545–564.

[32] López-García P, Kazmierczak J, Benzerara K, Kempe S, Guyot F, Moreira D. Bacterial diversity and carbonate precipitation in the giant microbialites from the highly alkaline Lake Van, Turkey. Extremophiles. 2011: 263–274. 10.1007/s00792-005-0457-0 15959626

[33] Kempe S, Kazmierczak J. Hydrochemical key to the genesis of calcareous nonlaminated and laminated cyanobacterial microbialites. Cellular Origin Life in Extreme Habitats & Astrobiology. 2007;11: 239–264.

[34] Komárek J, Anagnostidis K. Cyanoprokaryota. Spektrum Akademischer Verlag; 2000.

[35] Silva SMF, Pienaar RN. Benthic marine Cyanophyceae from Kwa-Zulu Natal, South Africa. Bibliotheca Phycologica 2000.

[36] Vandenbroucke M, Largeau C. Kerogen origin, evolution and structure. Organic Geochemistry. 2007;38(5): 719–833.

[37] Li J, Benzerara K, Bernard S, Beyssac O. The link between biomineralization and fossilization of bacteria: insights from field and experimental studies. Chemical Geology. 2013;359(1): 49–69.

[38] Schopf JW, Kudryavtsev AB, Czaja AD, Tripathi AB. Evidence of Archean life: stromatolites and microfossils. Precambrian Res. 2007;158 (3–4): 141–155.

[39] Golubic S, Hofmann HJ. Comparison of Holocene and Mid-Precambrian Entophysalidaceae (Cyanophyta) in Stromatolitic Algal Mats: Cell Division and Degradation. Journal of Paleontology. 1976;50(6): 1074–1082.

[40] Dove PM, Yoreo JJD, Weiner S. Biomineralization. Mineralogical Society of America: Washington, DC, USA; 2003.

[41] Dupraz C, Reid RP, Braissant O, Decho AW, Norman RS, Visscher, PT. Processes of carbonate precipitation in modern microbial mats. Earth Science Review. 2009;96: 141–162.

[42] Kamennaya NA, AjoFranklin CM, Northen T, Jansson C. Cyanobacteria as biocatalysts for carbonate mineralization. Minerals. 2012;2(4): 338–364.

[43] Miller AG, Espie GS, Canvin DT. Physiological aspects of CO_2_ and HCO3− transport by cyanobacteria: a review. Revue Canadienne De Botanique. 1990;68(6): 1291–1302.

[44] Kawaguchi T, Decho AW. A laboratory investigation of cyanobacterial extracellular polymeric secretions (EPS) in influencing CaCO_3_ polymorphism. Journal of Crystal Growth. 2002;240: 230–235.

[45] Dupraz C, Visscher PT, Baumgartner LK, Reid RP. Microbe–mineral interactions: early carbonate precipitation in a hypersaline lake (Eleuthera Island, Bahamas). Sedimentology. 2004;51: 745–765.

[46] Knauth L P, Kennedy M J. The late Precambrian greening of the Earth. Nature. 2009; 460(7256): 728–732. 10.1038/nature08213 19587681

[47] Mazzullo SJ. Organogenic dolomitization in peritidal to deep-sea sediments. Journal of Sedimentary Research. 2009;70(1): 10–23.

[48] Richards FA. Anoxic basins and fjords In Chemical Oceanography. Academic Press, London 1965: 623–645.

[49] Decho AD. Exopolymer microdomains as a structuring agent for heterogeneity within microbial biofilms In: Riding R.E., Awramik S.M. (Eds.), Microbial Sediments. Springer-Verlag; 2000 pp. 1–9.

[50] Paerl HW, Steppe TF, Reid RP. Bacterially mediated precipitation in marine stromatolites. Environmental Microbiology. 2001;3(2): 123–30. 11321542 10.1046/j.1462-2920.2001.00168.x

[51] Kremer B, Kazmierczak J, Stal LJ. Calcium carbonate precipitation in cyanobacterial mats from sandy tidal flats of the North Sea. Geobiology. 2008;6(1): 46–56. 10.1111/j.1472-4669.2007.00128.x 18380885

[52] Tazaki K. Biomineralization of layer silicates and hydrated Fe/Mn oxides in microbial mats: An electron microscopical study. Clays and Clay Minerals. 1997;45: 203–212.

[53] Ueshima M, Tazaki K. Possible role of microbial polysaccharides in nontronite formation. Clays and Clay Minerals. 2001;49: 292–299.

[54] Konhauser K. Introduction to Geomicrobiology. Blackwell Publishing, Malden, MA; 2007 pp. 425.

[55] Pace A, Bourillot R, Bouton A, Vennin E, Galaup S, Bundeleva I, et al Microbial and diagenetic steps leading to the mineralisation of Great Salt Lake microbialites. Scientific Reports.2016;6: 31495 10.1038/srep31495 27527125 PMC4985759

[56] Kremer B, Kaźmierczak J, Kempe S. Authigenic replacement of cyanobacterially precipitated calcium carbonate by aluminium-silicates in giant microbialites of Lake Van (Turkey). Sedimentology. 2019; 66(1): 285–304.

[57] Fein JB, Scott S, Rivera N. The effect of Fe on Si adsorption by *Bacillus subtilis* cell walls: insights into nonmetabolic bacterial precipitation of silicate minerals. Chemical Geology. 2002;182: 265–273.

[58] Riding R. Microbial carbonates: the geological record of calcified bacterial–algal mats and biofilms. Sedimentology. 2000;47(s1): 179–214.

